# Regulatory T Cells Compensation Failure Cause the Dysregulation of Immune Response in Pristane Induced Lupus Mice Model

**DOI:** 10.21315/mjms2018.25.3.3

**Published:** 2018-06-28

**Authors:** Handono Kalim, Mirza Zaka Pratama, Aditya Satriya Nugraha, Multi Prihartini, Afriska Chandra, Al Imroatus Sholihah, Fatina Qonita, Kusworini Handono

**Affiliations:** 1 Division of Rheumatology and Immunology, Department of Internal Medicine, Faculty of Medicine Brawijaya University/Dr. Saiful Anwar Hospital, Malang, Indonesia; 2 Faculty of Medicine, Brawijaya University, Malang, Indonesia; 3 Department of Clinical Pathology, Faculty of Medicine Brawijaya University, Malang, Indonesia

**Keywords:** systemic lupus erythematosus, regulatory T cells, pristane

## Abstract

**Introduction:**

Regulatory T cells’ (Tregs’) role remains unclear in the pathogenesis of systemic lupus erythematosus (SLE). This study was aimed at monitoring the percentage of Tregs within 32 weeks and monitoring its relationship with the percentage of other T helper (Th) cell subsets and the levels of autoantibodies and pro-inflammatory cytokines in a murine SLE model induced by pristane.

**Methods:**

Forty-eight female BALB/c mice were divided into a healthy control (HC) and a pristine-induced (PI) group. SLE was induced by a single 0.5 cc pristane intraperitoneal injection. Six from each group were sacrificed every eight weeks until 32 weeks post-pristane injection. Treg, Th1, Th2 and Th17 percentages from the spleen were measured using flowcytometry. ANA, IL-6 and IFN-α levels were measured from serum using ELISA.

**Results:**

The Treg percentage from the PI group increased significantly at 16 weeks compared to the HC group, while Th1, Th2 and Th17 percentages decreased. Tregs in the PI group began to reduce from the 24th to 32nd weeks, followed by an elevation of the Th1, Th2 and Th17 percentages. Tregs were negatively correlated with Th1 and Th2. Tregs in the PI group had a negative correlation with ANA and IFN-α levels from serum, whereas Tregs had a positive correlation with IL-6 levels.

**Conclusion:**

The compensation of Tregs observed at 16 weeks after pristane injection failed, marked by a decreasing number of Tregs, followed by an increase of Th subsets, pro-inflammatory cytokines and autoantibodies. This compensatory failure of Tregs could be affected by pro-inflammatory cytokines, such as IFN-α and IL-6.

## Introduction

Systemic lupus erythematosus (SLE) is a systemic autoimmune inflammatory disease that has a complex pathogenesis (1). Recent studies have discovered that the pathogenesis of SLE is correlated with the abnormal response of CD4^+^ T cells, including regulatory T cells (Tregs) and T helper (Th) cell subsets (2–4). Tregs keep peripheral tolerance acts by preventing autoimmune diseases, such as SLE (5). Defects in the Treg numbers and function have been reported in SLE and are associated with the disease progressivity and organ damages (6–8).

Until today, the role of Tregs in SLE was still controversial. Previous studies determined that there is a decrease in the number of Treg populations in SLE patients (6–8). A similar study also found that the decreasing number of Tregs in SLE is caused by their greater susceptibility to Fas-induced apoptosis than the healthy control (9). However, other findings show some contradicting results. Our previous study showed that there is a significant increase in Treg numbers in SLE patients in Indonesia compared to a healthy control (10). Similar phenomenon showing a significant increase in the Treg numbers were also shown in other studies (11–13). Despite these contradictory results of the role of Tregs in SLE, better knowledge about Tregs will not only provide new insights into the pathogenesis of SLE but also can be used to develop various clinical applications, including preventive and curative treatment for SLE.

Differences in the stage, activity, manifestation and therapy of the disease may influence the results that were found in the previous studies. Heterogeneities of SLE patients and ethical problems may also lead to difficulties in investigating the role of Tregs in SLE. Therefore, in our present study, we assessed the percentages of Tregs periodically and estimate the relationship of Tregs with the development of other Th cell subsets and the levels of autoantibodies and pro-inflammatory cytokines production in a homogenised murine model of SLE over 32 weeks. So, hopefully this study will answer the question regarding the role of Tregs in the pathogenesis of SLE.

## Methods

### Animals and SLE Induction

Forty-eight 8 to 12 week-old female BALB/c mice were purchased from Pusvetma Surabaya (East Java, Indonesia). All mice were kept under pathogen-free conditions and were provided with food and water ad libitum. All experiments had been approved by the ethical committee of Brawijaya University on the care and use of animals and with related codes of practice. After a week of acclimatisation, all mice were prepared for SLE induction. Mice were divided into two groups: a healthy control (HC) and pristine-induced (PI) group. The PI group was injected with 0.5 mL of pristane (Sigma, St Louis, MO) intraperitoneally, whereas the HC group were injected with phosphate-buffered saline (PBS) in the same manner. All injections were done only one at a time at the first time of the study.

### Measurement of Treg and Th Cell Subset Percentages

Every eight weeks, six mice from each group were killed in order to periodically monitor the Tregs and Th cell subsets. The Treg and Th cell subset percentages were measured from the spleen using flowcytometry. Cells that had been harvested from the spleen were stained using antibody markers. Treg detection was done by labelling with PE anti-mouse CD4 (Biolegend), PerCP anti-mouse (Biolegend) and FITC anti-mouse FoxP3 (Biolegend). Th cell subsets, including Th1, Th2 and Th17, were also measured in a similar manner. Prior to staining these cells, cells were stimulated with phorbol myristate acetate (PMA; 50 ng/mL; Sigma, St Louis, MO) and ionomycin (1 μg/mL; Sigma) in the presence of brefeldin A (BD Pharmingen, San Diego, CA) for at least four hours. Then, cells were labelled with PE anti-mouse CD4 (Biolegend). Intracellular staining was performed using PerCP anti-mouse IL-4 (Biolegend), FITC anti-mouse IFN-γ (Biolegend) and PerCP anti-mouse IL-17A to detect Th1, Th2 and Th17, consecutively. All staining was performed according to Biolegend manufacture protocols. All cells were analysed with FACSCalibur (Becton Dickinson).

### Measurement of Cytokines and Autoantibody Levels

Cytokine and autoantibody levels from each group were measured from serum every eight weeks. Cytokines IL-6 and IFNα were measured using an enzyme-linked immunosorbent assay (ELISA) with commercially available kits from Biolegend. Similarly, anti-nuclear antibody (ANA) levels were measured from serum using ELISA kits (MyBioSource, San Diego, CA). All ELISA measurements were done according to the manufacturer’s protocols. IL-6 is a key cytokine that can affect the function of Tregs, while IFNα has a suppressive ability against Treg function.

### Statistical Analysis

A comparison of the changes in Tregs and Th cell subsets, cytokines and ANA levels over time were done with a two-way analysis of variance (ANOVA), followed by Tukey’s post hoc test. The correlation between Tregs and other variables (Th1, Th2, Th17, ANA, IFNα and IL-6) were determined by Pearson analysis. Statistical analyses were performed using the GraphPad 6 software. A *P*-value less than 0.05 was considered significant.

## Results

### Development of Treg Percentages after Pristane Injection

**Figure 1:**
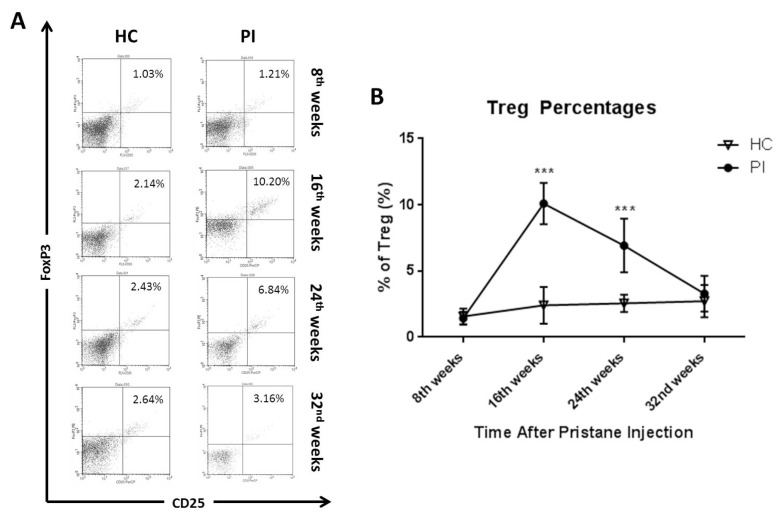
Profiles of Treg percentages on every eighth week. A: Representative figures of a dot plot flowcytometry analysis from each group from every eighth week. Tregs expressed CD4, CD25 and FoxP3, as shown in upper right quadrant. B: Mean of Treg percentage every eight weeks from the PI and HC group, data presented as mean ± SD with *P < 0.05, **P < 0.01 and ***P < 0.001 compared to the HC

To monitor the development of Treg percentages after pristane injection, we periodically evaluated Treg percentages from the spleens of the PI and HC mice every eight weeks using flowcytometry. Tregs were counted as cells that expressed CD4, CD25 and FoxP3, as shown in Figure 1A and Table 1. The progression of Treg percentages after pristane injection is shown in Figure 1B. At 8 weeks after injection, there were no statistically different Treg percentages in the PI and HC group (*P* > 0.999). Significant increases in the Treg percentages from the PI group were found at 16 weeks (*P* < 0.001) and 24 weeks (*P* < 0.001) after the pristane injection compared to the HC group. As seen in Figure 1B, the Treg percentage from the PI group gradually decreased at the 24th and 32nd weeks and were significantly different compared to the 16th week (*P* = 0.004 and *P* < 0.001, respectively). At the end of the 32nd week, there were no statistically significant differences in Treg percentages between the PI and HC groups (*P* = 0.996).

### Development of Th Cell Subset Percentages after Pristane Injection

**Figure 2:**
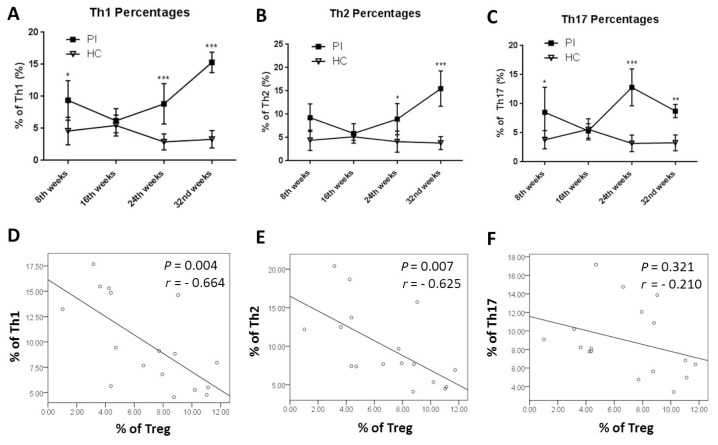
Profiles of Th cell percentages and correlation with Tregs. A–C: Mean of Th1, Th2 and Th17 percentages from the PI and HC groups from every eighth week. D–F: Correlation between Th1, Th2 and Th17 percentages with Treg percentage in the PI group. Data presented as mean ± SD with *P < 0.05, **P < 0.01 and ***P < 0.001 compared to the HC

We also monitored the development of the Th cell subset percentages, including Th1, Th2 and Th17, after pristane injection using a two-way analysis of variance (ANOVA) followed by a Tukey’s post hoc test. As shown in Figures 2A and 2C, at eight weeks after pristane injection, the Th1 and Th17 percentages were significantly higher in the PI group compared to the HC group (*P* = 0.022 and *P* = 0.027, respectively) but not the Th2 percentage (*P* = 0.072). Interestingly, there was some decrease in the Th1, Th2 and Th17 percentages at the 16th week for the PI group compared to the 8th week, as previously shown in Table 2. However, these reductions did not seem to be statistically significant compared to the 8th week (*P* = 0.297, *P* = 0.414 and *P* = 0.299, respectively). We also found that there were gradual increases in the Th1 and Th2 percentages after 24 and 32 weeks post-pristane injection, which were significantly different compared to the HC group (*P* < 0.001, *P* = 0.047, *P* < 0.001 and *P* < 0.001 for Th1 and Th2 at the 24th and 32nd weeks, respectively; Figures 2A–B). However, a different development was found in the Th17 percentages (Figure 2C). Similar to the other two, Th17 percentages increased significantly at 24 weeks post–pristane injection compared to the HC group (*P* < 0.001). However, a decrease in the Th17 percentage in the PI group was found at 32 weeks after pristane injection but was still significantly different compared to the HC (*P* = 0.003; Figure 2C).

From the correlation analysis using Pearson analysis, we found that there was a significant, negative correlation between the Th1 and Treg percentages (*P* = 0.004, *r* = −0.664; Figure 2D) as well as the Th2 and Treg percentages (*P* = 0.007, *r* = −0.625; Figure 2E), but Th17 was not significantly correlated with the Treg percentage (*P* = 0.321, *r* = −0.210; Figure 2F) in the PI group.

### Autoantibody and Pro-inflammatory Cytokine Levels

**Figure 3:**
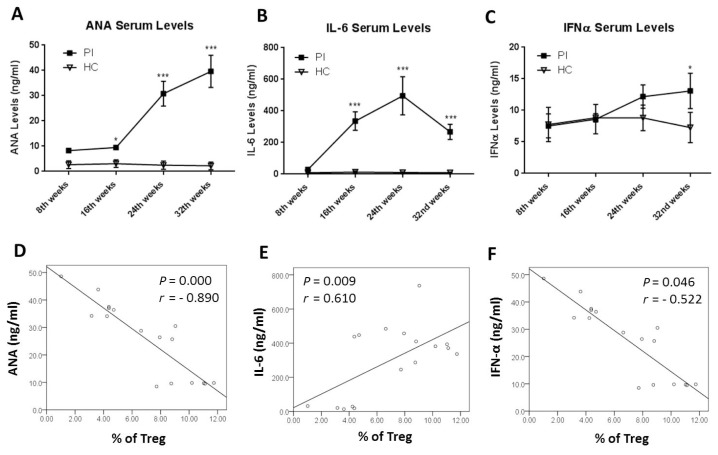
Autoantibody and pro-inflammatory cytokines levels with correlation to Treg percentages. A–C: Mean of ANA, IL-6 and IFN-α levels from the serum of the PI and HC groups from every eighth week. D–F: Correlation between ANA, IL-6 and IFN-α levels with Treg percentage in the PI group. Data presented as mean ± SD with *P < 0.05, **P < 0.01 and ***P < 0.001 compared to HC

In this study, by using a two-way analysis of variance (ANOVA) followed by Tukey’s post hoc test, we also observed the development of autoantibody production by measuring ANA level from the serum. From Figure 3A, we can see that there was no significant difference in ANA levels between the PI and HC group at the 8th week (*P* = 0.172). A gradual increase of ANA levels in the PI group with significant differences compared to the HC group was found at 16 weeks, 24 weeks and 32 weeks after pristane injection (*P* = 0.034, *P* < 0.001 and *P* < 0.001, respectively, as shown in Figure 3A and Table 3).

We also analysed the development of pro-inflammatory cytokine production by measuring IL-6 and IFN-α serum levels. As shown in Figure 3B, no significant difference was observed in IL-6 levels at the 8th week in the PI group compared to the HC group (*P* = 0.999). IL-6 levels in the PI group increased gradually and were statistically different compared to the HC group starting at the 16th week (*P* < 0.001) and were at their highest levels at 24 (*P* < 0.001) weeks after pristane injection (Figure 3B). After 32 weeks, IL-6 decreased significantly compared to 24 weeks post-injection in the PI group (*P* < 0.001; Figure 3B). The development of IFN-α production was different from IL-6 production, as shown in Figure 3C. IFN-α levels from the PI and HC group showed no significant differences at 8, 16 and 24 weeks after pristane injection (*P* > 0.999, *P* > 0.999 and *P* = 0.430, respectively). A significant increase of IFN-α levels from the PI group was found 32 weeks after pristane injection, as seen in Figure 3C (*P* = 0.006).

A correlation between autoantibody and pro-inflammatory cytokine production with Treg percentage in the PI group found by using Pearson analysis is shown in Figures 3D–F. There was a significant, negative correlation between the ANA level and Treg percentage (*P* = 0.000, *r* = −0.890; Figure 3D). We also found a significant, negative correlation between IFN-α level and Treg percentage (*P* = 0.046, *r* = −0.522; Figure 3F). Whereas a significant, positive correlation was found between IL-6 level and Treg percentage from the PI group (*P* = 0.009, *r* = 0.610; Figure 3E).

## Discussion

An abnormal number and function of Tregs is a new paradigm in the pathogenesis of SLE, which still has many controversies. Recent studies have found that there is some decrease in the number of Tregs in SLE patients, which correlates with poor prognosis in SLE (6–9). On the other hand, other studies determined that there is an increase in Treg numbers in SLE patients, indicating compensatory mechanisms against autoinflammatory processes in SLE (10–13). These contradictory results from these previous studies make the role of Tregs in SLE unclear. We observed that there were dynamic changes in Treg percentages found in a murine model of SLE. There was a significant increase in Treg percentages after 16 weeks post-pristane injection, followed by significant decrease 24 and 32 weeks after injection. Our findings confirmed that Tregs could both increase and decrease in SLE depending on the onset time of the disease.

The increase in Treg percentages at 16 weeks after pristane injection was followed by decreases in Th1, Th2 and Th17 percentages from the 8th to 16th weeks post-pristane injection (Figure 2). This result indicates that there may be a compensatory mechanism of Treg against other Th cell subsets in the early onset of the disease in a murine model of SLE. Our findings are also consistent with another study conducted in SLE patients. Bonelli et al. observed that CD4^+^ CD25^+^ Tregs increase in patients with newly onset SLE (14). Interestingly, Zhang et al. also found that Treg numbers increase in patients with newly onset SLE, have normal suppressive functions and positively correlate with anti-dsDNA titers, indicating a compensatory mechanism against autoimmunity (15).

At later onsets, our findings showed that the decrease in Treg percentage was followed by an increase in Th cell subsets, including Th1, Th2 and Th17. This decrease was also negatively correlated with Th1 and Th2 percentages. In addition, we also found that there was a strong inverse correlation between ANA levels and Treg percentages (Figure 3D). These data raise a speculation that there may be some defects in Treg function resulting in the failure of Treg compensation in the later onset of SLE. This can lead to an increase of Th cell subsets and an enhancement of autoantibody production. Our latest study revealed that there is a decrease in the migratory function of Tregs in SLE patients despite the fact that some increase in the number of Tregs is also found in the study (16). In another study, we also determined that there is a decrease of Treg secretory function as shown by a decrease of TGF-β1 levels in SLE patients (10). Similar results were also observed in other studies, where there is a decrease of suppressive ability of Tregs in SLE patients in modulating other Th cell subsets (13, 17).

The composition of the local milieu, including the types of cytokines, can influence Treg function (18). Some pro-inflammatory cytokines, such as IFN type I (IFN-α) and IL-6 have been found to affect Treg function in SLE patients (19, 20). Our findings showed that there was a negative correlation between Treg percentage and IFN-α level, indicating the possibility of a suppressive ability of IFN-α against Treg function (Figure 3F). However, the mechanisms of immune modulation by IFN-α remain not clearly understood. Previous studies determined that antigen-presenting cell (APC) activation by IFN-α can decrease Treg function and enhance Th cell functions (11, 21). Similarly, Bacher et al. demonstrated that IFN-α can deactivate the suppressive function of human Tregs by downregulating their intracellular cyclic adenosine monophosphate (cAMP) level (22). Interestingly, we showed that IL-6 highly increased in a murine model of SLE and positively correlated with Treg percentages. Recent studies determined that IL-6 is a key cytokine that can reduce the function of Tregs (20, 23). However, it remains controversial whether excessive production of IL-6 can affect the function of Tregs. An interesting study done by Fujimoto et al. shows that excessive IL-6 production in IL-6 transgenic mice can increase the number of Tregs (24). Thus, it still may be possible that the elevation of IL-6 levels is the potential cause of Tregs’ increase in order to compensate for pro-inflammatory conditions in SLE. However, further research still needs to be done to prove this hypothesis.

Our current study provides some new information regarding to the role of Tregs in the pathogenesis of SLE. Different onsets of the disease may raise different manipulation methods to preserve Treg numbers and function in SLE. Even though we demonstrated that there were dynamic changes in Treg numbers in a murine model of SLE, we did not observe changes of Treg function throughout the study. We also found that some pro-inflammatory cytokines could affect Treg numbers. However, it is possible that not only can IL-6 and IFN-α affect Tregs but the other cytokines perhaps also have roles in modulating Tregs in SLE. Although many limitations can be found in the study, further research to cover these limitations are still possible to be done in the future so that the role of Tregs in SLE can be clearly understood.

## Conclusion

In summary, this study demonstrated that Treg numbers in a murine model of SLE can both increase and decrease depending on the onset time of the disease. An early increase of Tregs is considered to be a compensatory mechanism against inflammatory response, including Th cell subsets. A decrease in the number of Tregs with a later onset of the disease negatively correlates with Th cell subsets’ numbers and autoantibody levels, which indicates a compensatory failure mechanism of Tregs. This compensatory failure of Tregs was found to be affected by pro-inflammatory cytokines, such as IFN-α and IL-6. Thus, this research provides some new insights regarding the role of Tregs in SLE.

## Figures and Tables

**Table 1:** Comparison of Treg percentages between groups

Treg percentages (%)	HC	P*	PI	P*	*P*#
8th Weeks	1.57 ± 0.59	-	1.43 ± 0.49	-	> 0.999
16th Weeks	2.40 ± 1.39	0.947	10.09 ± 1.56	< 0.001	< 0.001
24th Weeks	2.56 ± 0.65	> 0.999	6.92 ± 2.03	0.003	< 0.001
32th Weeks	2.72 ± 1.22	0.996	3.26 ± 1.36	0.001	0.996

* *P*-value showed the comparison between different times in the same group (HC or PI), compared to previous weeks

# *P*-value showed the comparison between HC and PI group in the same week

**Table 2:** Comparison of Th cell percentages between groups

	HC	P*	PI	P*	*P*#
Th1 Percentages (%)					
8th Weeks	4.59 ± 2.16	-	9.37 ± 3.08	-	0.022
16th Weeks	5.45 ± 1.65	0.996	6.19 ± 1.88	0.297	0.999
24th Weeks	2.88 ± 1.24	0.482	8.83 ± 3.15	0.385	0.001
32th Weeks	3.30 ± 1.36	> 0.999	15.31 ± 1.60	0.003	< 0.001
Th2 Percentages (%)					
8th Weeks	4.41 ± 2.17	-	9.27 ± 2.96	-	0.072
16th Weeks	5.16 ± 0.77	0.999	5.88 ± 2.10	0.414	0.997
24th Weeks	4.12 ± 2.27	0.997	8.95 ± 3.34	0.397	0.047
32th Weeks	3.81 ± 1.38	> 0.999	15.50 ± 3.79	0.002	< 0.001
Th17 Percentages (%)					
8th Weeks	3.80 ± 1.58	-	8.53 ± 4.29	-	0.027
16th Weeks	5.60 ± 1.81	0.819	5.33 ± 1.23	0.299	> 0.999
24th Weeks	3.18 ± 1.43	0.515	12.80 ± 3.17	< 0.001	< 0.001
32th Weeks	3.27 ± 1.37	> 0.999	8.74 ± 1.13	0.057	0.003

* *P*-value showed the comparison between different times in the same group (HC or PI), compared to previous weeks

# *P*-value showed the comparison between HC and PI group in the same week

**Table 3:** Comparison of autoantibody and pro-inflammatory cytokines levels between groups

	HC	P*	PI	P*	*P*#
ANA (ng/mL)					
8th Weeks	2.68 ± 1.54	-	8.25 ± 0.65	-	0.172
16th Weeks	3.08 ± 1.53	> 0.999	9.48 ± 0.50	0.999	0.0034
24th Weeks	2.46 ± 1.62	> 0.999	30.80 ± 4.88	< 0.001	< 0.001
32th Weeks	2.22 ± 1.49	> 0.999	39.64 ± 6.37	0.001	< 0.001
IL-6 (ng/mL)					
8th Weeks	9.32 ± 4.02	-	29.03 ± 11.80	-	0.999
16th Weeks	12.88 ±7.77	> 0.999	445.73 ± 58.81	< 0.001	< 0.001
24th Weeks	10.58 ± 3.92	> 0.999	495.55 ± 120.69	0.006	< 0.001
32th Weeks	9.32 ± 4.02	> 0.999	267.40 ± 48.14	< 0.001	< 0.001
IFNα (ng/mL)					
8th Weeks	7.75 ± 2.73	-	7.53 ± 1.89	-	> 0.999
16th Weeks	8.83 ± 0.66	0.995	8.60 ± 2.32	0.995	> 0.999
24th Weeks	8.80 ± 2.03	> 0.999	12.18 ± 1.85	0.255	0.430
32th Weeks	7.28 ± 2.40	0.970	13.08 ± 2.80	0.999	0.006

* *P*-value showed the commparison between different times in the same group (HC or PI), compared to previous weeks

# *P*-value showed the comparison between HC and PI group in the same week
